# Balanced crystalloids versus normal saline for initial fluid resuscitation in diabetic ketoacidosis: a systematic review and meta-analysis of randomized controlled trials

**DOI:** 10.62838/jccm-2026-0022

**Published:** 2026-07-27

**Authors:** Khaled Ahmed Reda Soliman, Ahmed Osman Hassan Ali, Farrukh Ameer, Bashaer Alharbi, Mohamed Hany Elmasry, Rawabi Mohammed Alwashmi, Bandar Saad Alshreef, Mohammad Saleh H Almabouth, Kinda Ahmed Hardan, Aymen Ali Alqurain

**Affiliations:** Department of Emergency Medicine, Armed Forces Hospital Southern Region, Khamis Mushait, Saudi Arabia; Department of Critical Care Medicine, Dr. Soliman Fakeeh Hospital, Riyadh, Saudi Arabia; Department of Adult Cardiology, Dr. Soliman Fakeeh Hospital, Fakeeh Care Group, Riyadh, Saudi Arabia; Department of Clinical Pharmacy, Ministry of Health, Jeddah, Saudi Arabia; Department of ICU and Anesthesia, Care Medical Hospital Almalaz, Riyadh, Saudi Arabia; Burydah College,Qassim, Saudi Arabia; Healthcare Management, College of Applied Medical Sciences, Shaqra University, Shaqra, Saudi Arabia; Department of Internal Medicine, NSH, Medina, Saudi Arabia; Senior House Officer of General Medicine, International University of Africa, Riyadh, Saudi Arabia; Department of Clinical Practice, Faculty of Pharmacy, Northern Border University,Saudi Arabia

**Keywords:** diabetic ketoacidosis, fluid therapy, fluid resuscitation, balanced crystalloids, normal saline

## Abstract

**Objective:**

To systematically synthesize evidence from RCTs that evaluate the efficacy and safety of balanced crystalloids compared with normal saline for initial fluid resuscitation of patients with DKA.

**Methods:**

This systematic review and meta-analysis were performed considering PRISMA guidelines and was registered in PROSPERO. A comprehensive search was performed to identify RCTs comparing balanced crystalloids with normal saline in adults and children with DKA. The risk of bias was assessed by using Cochrane RoB 2 tool. A random-effects meta-analysis was performed using R software to calculate pooled Mean Differences for continuous outcomes and Odds Ratios for dichotomous outcomes with 95% Confidence Intervals.

**Results:**

Eleven RCTs were included. In the quantitative synthesis of six RCTs (n = 491) using continuous time-to-event data, balanced crystalloids were not associated with a statistically significant reduction in time to DKA resolution compared with normal saline (Mean Difference [MD] = −1.50 hours; 95% CI: −3.79 to 0.79; p=0.15), with moderate heterogeneity (I2 = 36.2%). The 95% prediction interval ranged from −5.44 to 2.44 hours. However, balanced crystalloids resulted in a significantly greater increase in serum bicarbonate at 12 hours (MD = +2.50 mmol/L; 95% CI: 1.51 to 3.48; p=0.004; I2 = 0.0%). Subgroup analyses by fluid type, DKA severity, and age group showed no significant subgroup differences.

**Conclusion:**

Initial fluid resuscitation with balanced crystalloids was not associated with a shorter time to DKA resolution compared with normal saline, and they were associated with a rapid increase in serum bicarbonate levels; however, this biochemical improvement did not translate into a shorter time to DKA resolution or other clinical benefits. The choice of crystalloids for initial DKA resuscitation remains an area of clinical equipoise because of the substantial heterogeneity and methodological limitations of the available evidence, emphasizing the need for further high-quality research.

## Introduction

Diabetic ketoacidosis (DKA) is a life-threatening acute metabolic complication of diabetes mellitus that is characterized by the pathophysiological triad of hyperglycemia, ketosis, and high anion gap metabolic acidosis [1]. It arises from relative or absolute insulin deficiency, which is accompanied by an excess of counter-regulatory hormones, leading to intravascular volume depletion and electrolyte derangement [2]. Intravenous fluid resuscitation is the cornerstone of initial management, as it restores circulatory volume, improves tissue perfusion, and enhances the renal clearance of glucose and ketones, thereby aiding in the correction of–the base disturbances [3, 4].

The standard of care for initial fluid resuscitation has been the administration of 0.9% sodium chloride, referred to as normal saline [1], because of its historical precedent and effectiveness in expanding intravascular volume. However, its composition is non-physiological, containing a chloride concentration of 154 mmol/L, which exceeds that of human plasma [5]. The administration of large volumes of this chloride-rich fluid induces a non-anion gap and hyperchloremic metabolic acidosis [6, 7]. Iatrogenic acidosis may confound the biochemical resolution of DKA by sustaining low serum bicarbonate levels, thereby complicating the clinical assessment of recovery, and delaying the resolution of ketoacidosis [8]. In addition, an excessive chloride load has been associated with renal vasoconstriction and an increased risk of acute kidney injury (AKI) in critically ill populations [9, 10].

In response to the physiological concerns associated with normal saline, balanced crystalloid solutions, such as Lactated Ringer’s solution and plasma-Lytes, have emerged as compelling alternatives. These fluids possess a more physiological composition, with lower chloride concentrations and the inclusion of buffer anions such as lactate or acetate, which are metabolized to bicarbonate, thereby mitigating the risk of hyperchloremic metabolic acidosis [3].

It has been hypothesized that the use of balanced crystalloids may mitigate the development of hyperchloremic metabolic acidosis, leading to rapid normalization of biochemical parameters and faster resolution of DKA episodes [8, 11]. This hypothesis has been investigated in several randomized controlled trials (RCTs), but the evidence remains inconclusive. Some studies have suggested that balanced crystalloids lead to a more rapid resolution of acidosis and a shorter time to DKA resolution, whereas others have failed to demonstrate a difference in these or other patient-important outcomes [8, 12, 13]. Many of these trials were underpowered to detect robust differences in major clinical outcomes such as length of hospital stay, admission to the intensive care unit (ICU), or mortality. The heterogeneity in study designs, patient populations (pediatric vs. adult), and choice of balanced crystalloids have contributed to clinical requirements [14]. As a result, major clinical practice guidelines remain equivocal, offering no definitive recommendation for one crystalloid type over another for the initial resuscitation of DKA [4].

Given the uncertainty and high incidence of DKA, a definitive synthesis of the available evidence is imperative to inform clinical practice. Therefore, the primary objective of this systematic review and meta-analysis (SRMA) is to synthesize evidence from all available RCTs to compare the efficacy of balanced crystalloids versus normal saline for initial fluid resuscitation in patients with DKA, specifically to assess the time to resolution of DKA. Secondary objectives included a comparative analysis of the effects on the time to normalization of acidosis, incidence of hyperchloremia, requirements for insulin therapy, and major clinical outcomes, including length of hospital stay, ICU admission, and mortality.

## Materials and Methods

### Protocol and Registration

This SRMA was conducted and reported in accordance with the Preferred Reporting Items for Systematic Reviews and Meta-Analyses (PRISMA) guidelines [15]. The protocol was registered in the International Prospective Register of Systematic Reviews (PROSPERO) under registration number CRD420251157001 [16].

### Eligibility criteria

Studies were considered for inclusion based on a pre-specified Population, Intervention, Comparator, Outcomes, and Study Design (PICOS) framework. Eligible studies were RCTs with parallel-group and cluster-randomized designs. The study population included patients of any age or sex who presented with a clinical and biochemical diagnosis of DKA in an acute care setting. The intervention of interest was the use of any balanced buffered crystalloid solution (e.g. Ringer’s lactate, Plasma-Lyte, Hartmann’s solution) for the initial fluid resuscitation. Normal saline was used as a comparator for the same purpose. Studies were excluded if they focused on other forms of ketoacidosis, used balanced crystalloids only for maintenance therapy rather than initial resuscitation, or had a nonrandomized design.

### Information sources and search strategy

A comprehensive and systematic search of several major bibliographic databases was performed from their inception to 2025. The databases included the Cochrane Central Register of Controlled Trials (CENTRAL), MEDLINE (via PubMed), Embase, CINAHL, and Scopus. In addition, clinical trial registries ClinicalTrials.gov and the WHO International Clinical Trials Registry Platform (ICTRP) were searched to identify ongoing or recently completed trials. The search strategy combined Medical Subject Headings (MeSH) terms and keywords related to “diabetic ketoacidosis”, “fluid therapy”, “resuscitation”, “balanced solutions”, “Ringer’s lactate”, and “normal saline”. No date or language restrictions were applied in the search, and only studies published in English were included finally as non-English studies were excluded during the initial screening stage. To ensure the retrieval of relevant literature, backward and forward citation searches of the included articles and relevant reviews were performed manually.

### Study selection

The initial database search yielded 948 records. All retrieved citations were imported into reference management software, and 278 duplicates were removed. After screening the titles and abstracts of the remaining 670 records for eligibility, 550 were excluded as they did not meet the inclusion criteria. Subsequently, the full texts of the remaining 120 reports were retrieved, of which 32 were obtained and assessed for eligibility according to the PICOS criteria. Disagreements at either the abstract or full-text screening stage were resolved through discussion and adjudication by a third reviewer. After the full-text review, 21 reports were excluded for specific reasons, including insufficient data (n=10), lack of a control group (n=6), non-RCT design (n=3), and irrelevant patient populations (n=2), culminating in the final inclusion of 11 studies for qualitative and quantitative synthesis.

### Data extraction

A standardized data extraction form was developed and piloted prior to use, and relevant data were extracted from the included studies, including study characteristics (e.g., first author, year of publication, study design, country, and sample size), patient demographics (e.g., age, sex, and severity of DKA at presentation), intervention and comparator details (e.g., type and volume of fluid administered), and all pre-specified primary and secondary outcome measures. Data on the primary outcome, time to DKA resolution (Supplementary material Table S1), and secondary outcomes such as time to acidosis normalization, incidence of hyperchloremia, length of hospital stay, and mortality (Supplementary material Table S2) were extracted.

### Risk of bias assessment

The methodological quality and risk of bias for each included RCT were independently assessed by two reviewers using the revised Cochrane risk-of-bias tool for randomized trials (RoB 2) [17]. Each study was evaluated across five distinct domains: bias arising from the randomization process, bias due to deviations from the intended interventions, bias due to missing outcome data, bias in the measurement of the outcome, and bias in the selection of the reported result. Each domain was judged as “low risk of bias,” “some concerns,” or “high risk of bias,” leading to an overall risk of bias judgment for each study, whereas discrepancies in assessment were resolved through discussion.

### Data synthesis and statistical analysis

All statistical analyses were performed using the R statistical programming environment (version 4.51) [18], using the ‘meta’ and ‘metafor’ packages [19, 20]. The random-effects model proposed by DerSimonian and Laird was employed for all meta-analyses to account for the anticipated clinical and methodological heterogeneity among the included studies [21]. The Hartung-Knapp-Sidik-Jonkman adjustment for random-effects models was utilized to provide more conservative confidence intervals (CIs) because of the small number of studies. Prediction intervals were calculated to estimate the range in which the effect of a future study would fall.

For dichotomous outcomes, such as the incidence of hyperchloremia or ICU admission, pooled Risk Ratios (RRs) with corresponding 95% CIs were calculated. For continuous outcomes measured on the same scale, such as time to DKA resolution and length of hospital stay, the mean difference (MD) with 95% CIs was pooled. Statistical heterogeneity across studies was quantified using the I^2^ statistic, where values of 25%, 50%, and 75% were interpreted to represent low, moderate, and high levels of heterogeneity, respectively [22]. The statistical significance of heterogeneity was assessed using Cochran’s Q test, with a p-value less than 0.10 considered indicative of significant heterogeneity. The results of the meta-analysis were presented using forest plots. A narrative synthesis of the findings was conducted in cases where substantial heterogeneity (I^2^ > 75%) rendered statistical pooling inappropriate. The potential for publication bias was assessed by visual inspection of funnel plots for asymmetry and was tested using Egger’s regression test, contingent upon the inclusion of at least 10 studies in each meta-analysis [23]. The certainty of evidence for key outcomes was assessed using the Grading of Recommendations Assessment, Development and Evaluation (GRADE) approach.

## Results

### Study selection

**Figure 1.**
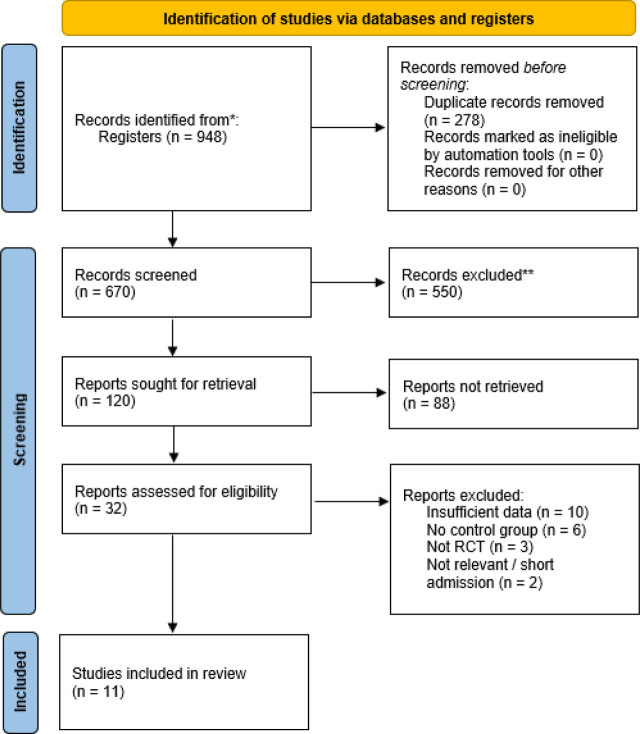
PRISMA 2020 flow diagram of study selection

The selection process culminated in the inclusion of 11 RCTs that met all the eligibility criteria for qualitative and quantitative synthesis in this systematic review (**Figure 1**).

### Characteristics of included studies

The 11 included RCTs represented a diverse collection of research enrolling participants across adult and pediatric populations that were published between 2011 and 2025, while five studies provided data on a total of 434 participants for the primary outcome of time to DKA resolution. The patient population was heterogeneous, with some evidence derived from pediatric cohorts. For the primary outcome analysis, three of the five studies focused on pediatric patients, whereas the remaining two focused on adults. The interventions across the studies involved the use of a balanced crystalloid solution, including Ringer’s lactate, Plasma-Lyte, and Hartmann’s solutions, which were compared with the control intervention of normal saline. The specific definitions of DKA and its resolution as well as baseline patient characteristics varied across trials. A comprehensive summary of the characteristics of the included studies is shown in Table 1.

**Table 1.** Characteristics of Included Studies

**Study**	**Country**	**Study design**	**Population**	**Intervention group (N)**	**Control group (N)**	**Primary outcome**	**Key findings**
Aditianingsih et al. (2017) [24]	Indonesia	Single-blind RCT	Adults with DKA	Ringerfundin (15)	NS (15)	Standard Base Excess (SBE) and Strong Ion Difference (SID).	BES resulted in higher SBE and SID but was not significantly superior to NS. Noted significant baseline imbalance in SID.
Agarwal et al. (2025) [25]	India	Double-blind RCT	Pediatric (9 mo-12 yrs) with DKA	RL (33)	NS (34)	Time to DKA resolution.	Time to resolution of DKA was shorter in the RL group compared to the NS group.
Attokaran et al. (2023) [26]	Australia	Nested cohort within an open-label, cluster-crossover RCT	Adults with DKA in the ED	PL (46)	NS (38)	Proportion of patients requiring ICU admission.	No significant difference in ICU admission rates. Noted very low compliance (40%) with the allocated PL fluid.
Mahler et al. (2011) [27]	USA	Double-blind RCT	Adults with moderate-to-severe DKA	PL (22)	NS (23)	Prevention of hyperchloremic metabolic acidosis.	PL prevented hyperchloremic metabolic acidosis and resulted in higher serum bicarbonate levels compared to NS.
Ramanan et al. (2021) [8]	Australia	Open-label, cluster-crossover RCT (SCOPE-DKA)	Adults with severe DKA admitted to ICU	PL (48)	NS (42)	DKA resolution defined as base excess ≥ −3 mEq/L at 48 hours.	PL led to a faster resolution of metabolic acidosis at 24 hours compared to NS.
Self et al. (2020) [12]^1^	USA	Pre-planned subgroup analysis of two cluster-crossover RCTs (SMART & SALT-ED)	Adults with DKA in the ED or ICU	BC (94)	NS (78)	Time to DKA resolution.	Treatment with BC resulted in a more rapid resolution of DKA compared with NS.
Trifi et al. (2025) [28]	Tunisia	Open-label RCT	Adults with severe DKA admitted to ICU	RL (46)	NS (42)	Composite of DKA resolution at 48 hours.	No significant difference in the rate of DKA resolution at 48 hours between the RL and NS groups.
Van Zyl et al. (2012) [13]	South Africa	Double-blind RCT	Adults with DKA	RL (28)	NS (29)	Time to pH normalization (pH ≥ 7.32).	The study failed to demonstrate a benefit from RL. The trial was stopped early due to slow enrolment.
Williams et al. (2020) [29]	India	Double-blind RCT (SPinK trial)	Pediatric (>1 mo- 12 yrs) with DKA	PL (34)	NS (32)	Incidence of new or progressive Acute Kidney Injury (AKI).	No significant difference in the incidence of AKI or the time to DKA resolution between groups.
Yan et al. (2024) [11]	Canada	Triple-blind pilot RCT (BRISK-ED trial)	Adults with DKA in the ED	RL (25)	NS (27)	Feasibility (recruitment rate) and efficacy (time to DKA resolution).	The trial protocol was feasible. Efficacy results showed a non-significant trend favouring NS for faster DKA resolution.
Yung et al. (2017) [30]	Australia	Double-blind RCT	Pediatric with moderate-to-severe DKA	HS (38)	NS (39)	Time to plasma bicarbonate ≥ 15 mmol/L.	No overall difference in outcomes, but a subgroup analysis suggested faster resolution of acidosis in patients with severe DKA.

Abbreviations: BC, Balanced Crystalloid; DKA, Diabetic Ketoacidosis; ED, Emergency Department; HS, Hartmann’s Solution; ICU, Intensive Care Unit; N, Number of participants; NS, Normal Saline (0.9% Sodium Chloride); PL, plasma lactate; RCT, randomized controlled trial; RL, Ringer’s lactate.

1 Self et al. performed a prespecified subgroup analysis of patients with DKA from the larger SMART and SALT-ED trials [12].

**Table 2.** Definitions of DKA Resolution in Included Studies

**Study**	**Definition of DKA Resolution**
Agarwal et al. (2025) [25]	pH >7.3 OR Bicarbonate >15, Ketones <2, Anion Gap <12
Self et al. (2020) [12]	Glucose <200 mg/dL + 2 of: Bicarbonate ≥15, pH >7.3, Anion Gap ≤12
Williams et al. (2020) [29]	pH >7.3, Bicarbonate >15, normal sensorium
Yan et al. (2024) [11]	Glucose <200 + 2 of: Bicarbonate ≥15, pH >7.3, Anion Gap ≤12
Yung et al. (2017) [30]	Bicarbonate >15 mmol/L (Biochemical only)
Van Zyl et al. (2012) [13]	pH ≥ 7.32 (Biochemical only)

### Risk of bias assessment

**Figure. 2.**
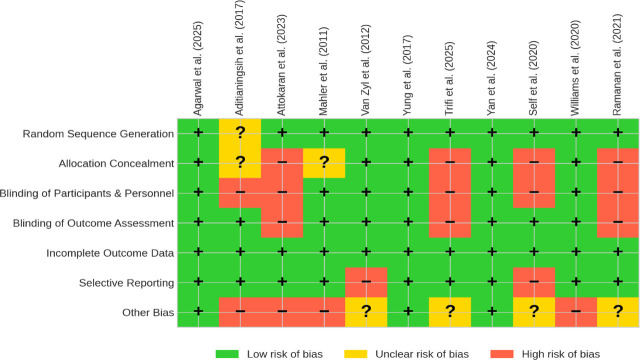
Risk of bias summary for included studies

**Figure. 3.**
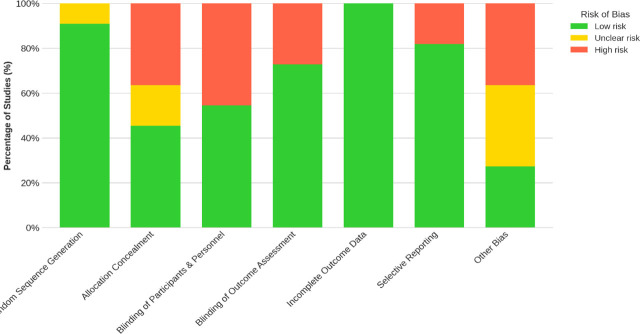
Risk of bias graph: Review authors’ judgements

The methodological quality of the 11 included studies was variable, with the overall risk of bias ranging from low to high across different domains. A detailed summary of the risk of bias assessment for each study is presented in Figure 2, and a summary of the risk of bias judgments across all the included studies is presented in Figure 3. The most prominent sources of potential bias were related to the blinding of participants and personnel, as several trials were open-label, and the” Other Bias’ domain, primarily due to significant baseline imbalances in key prognostic variables.

Although several studies have demonstrated a robust methodology with a low risk of bias in sequence generation and allocation concealment, a principal source of potential bias arose from the lack of blinding. Multiple trials were conducted in an open-label or single-blind manner, which introduces a high risk of performance bias, as clinician care may be influenced by knowledge of the treatment allocation. For objective laboratory measurements such as serum bicarbonate or chloride levels, the risk of detection bias was judged to be low. However, for outcomes involving clinical judgment, such as the decision for ICU admission or timing of discharge, the open-label design presented a high risk of detection bias. Furthermore, other sources of bias were identified in several studies. These included significant baseline imbalances between treatment groups, mostly in serum chloride and strong ion differences, which could confound the interpretation of the results. Additional concerns included low protocol compliance in the intervention arm of one cluster trial, leading to significant treatment contamination, and premature termination of one study, which introduces the risk of an underpowered or overpowered result. Conversely, bias due to incomplete outcome data and selective reporting of results was assessed as low-risk across most of the included trials.

### Meta-analysis of outcomes

#### Primary outcome: Time to DKA resolution

**Figure 4.**
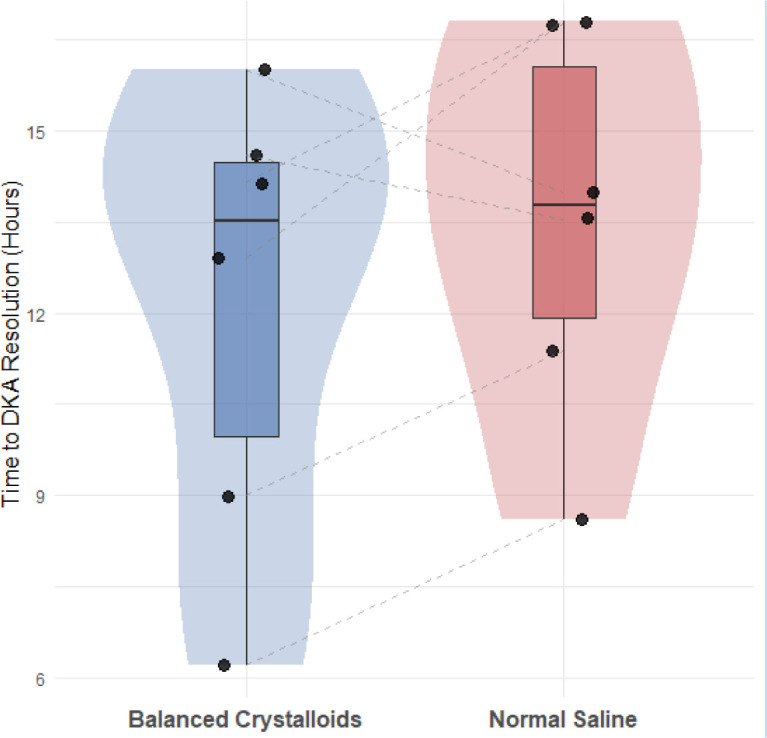
Distribution of study means for time to DKA resolution

The primary outcome was analysed by pooling data from six RCTs [11,12,13,25,29,30] encompassing 491 participants. The distribution of the mean resolution times from these studies is shown in Figure 4, illustrating the spread of the data. While most individual studies show a downward slope (faster resolution) toward the balanced crystalloid group, the overlapping distributions highlight clinical heterogeneity.

**Figure. 5.**
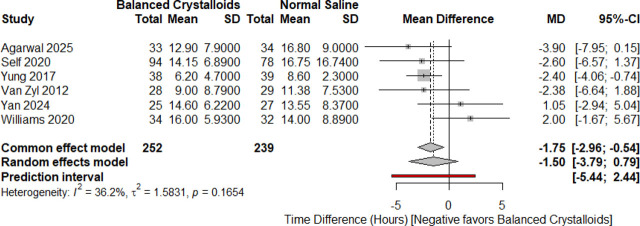
Forest plot of the mean difference in time to DKA resolution. The prediction interval (red line) estimates the range in which the true effect of the treatment is expected to lie in a future similar study, highlighting the potential for both benefit and harm in clinical practice.

The random-effects meta-analysis showed that balanced crystalloids were associated with a mean reduction of 1.50 hours in time to DKA resolution compared to normal saline, but this difference was not statistically significant (MD = −1.50 hours; 95% CI: −3.79 to 0.79; p = 0.15). Heterogeneity was moderate (I^2^ = 36.2%). The 95% prediction interval was wide (−5.44 to 2.44 hours), crossing the null effect, which indicates that in a future study, balanced crystalloids could either reduce resolution time by up to 5.4 hours or prolong it by 2.4 hours. Two studies [8, 28] reported resolution as a binary outcome at 48 hours and could not be pooled in the continuous analysis. A separate meta-analysis of these studies showed no significant difference in the odds of resolution at 48 hours (OR = 1.75; 95% CI: 0.68–4.48; p = 0.25) (Figure 5).

#### Secondary Outcomes

**Figure. 6.**
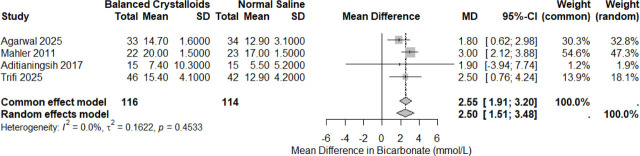
Forest plot of the odds ratio for the change in serum bicarbonate

**Change in serum bicarbonate at ∼12 h:** Data from four studies (n = 230) were pooled to assess biochemical recovery. Patients receiving balanced crystalloids demonstrated a significantly greater increase in serum bicarbonate levels at approximately 12 hours compared to those receiving normal saline (MD = +2.50 mmol/L; 95% CI: 1.51 to 3.48; p = 0.004) (Figure 6). No statistical heterogeneity was observed for this outcome (I^2^ = 0.0%), suggesting a consistent physiological effect across different trial settings.

**Hospital length of stay:** Four studies comprising 284 participants reported the length of hospital stay. Pooled analysis showed a non-significant trend towards a shorter hospital stay in the balanced crystalloid group (MD = −0.78 days; 95% CI: −2.35 – 0.79; p = 0.23). Moderate, although not statistically significant, heterogeneity was observed among the studies (I^2^ = 45.3%; p = 0.14).

**Figure. 7.**
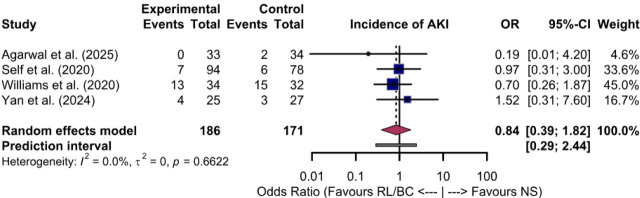
Forest plot of the odds ratio for the incidence of AKI

**Incidence of AKI:** A meta-analysis of four studies, including 357 participants, on the incidence of AKI revealed no significant difference between the two fluid groups. The pooled odds of developing AKI were 16% lower in the balanced crystalloid group, although this effect was not statistically significant (Odds Ratio [OR] = 0.84; 95% CI: 0.39 – 1.82; p = 0.52). No statistical heterogeneity was detected among the studies (I^2^ = 0.0%) (Figure 7).

**Incidence of hypokalemia:** Three studies with 258 participants were pooled to assess the incidence of hypokalemia (defined as serum potassium <3.5 mmol/L), which indicated a trend towards a protective effect with balanced crystalloids, where the odds of developing hypokalemia were 51% lower compared to normal saline, which approached, but did not reach, the conventional threshold for statistical significance (OR = 0.49; 95% CI: 0.23 – 1.02; p = 0.057), while no heterogeneity was observed for this outcome (I^2^ = 0.0%).

**ICU admission:** A meta-analysis of three studies, including 288 participants, evaluated the odds of ICU admission. The pooled estimate suggested a 39% reduction in the odds of ICU admission with the use of balanced crystalloids, but the result was not statistically significant and was characterized by a very wide confidence interval (OR = 0.61; 95% CI: 0.11 – 3.48; p = 0.58). The analysis was marked by substantial and statistically significant heterogeneity (I^2^ = 67.2%; p = 0.05), suggesting inconsistent effects across different clinical settings.

### Robustness and sensitivity analyses

#### Subgroup analysis

**Figure. 8.**
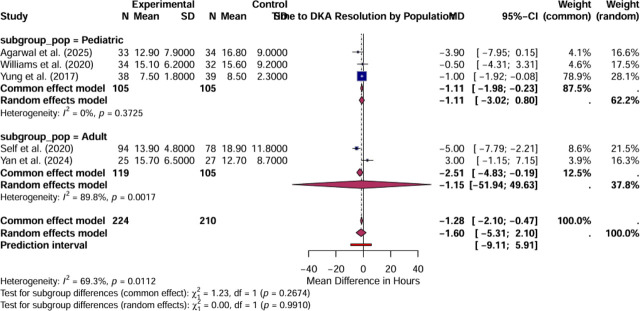
Subgroup analysis of time to DKA resolution by patient population (Pediatric vs. Adult). Prediction intervals are displayed to indicate the expected range of effects for future individual studies within each subgroup.

A prespecified subgroup analysis was conducted for the primary outcome of time to DKA resolution, stratifying studies by patient population (pediatric vs. adult). In the pediatric subgroup (three studies), balanced crystalloids were associated with a non-significant reduction in resolution time (MD = −1.11 hours; 95% CI: −3.02 – 0.80) with no heterogeneity observed (I^2^ = 0.0%). In the adult subgroup (two studies), the pooled effect estimate was similar (MD = −1.15 h), but the confidence interval was wide due to very high and statistically significant heterogeneity (I^2^ = 89.8%; p = 0.0017). A formal test for subgroup differences found no statistical evidence of an interaction between patient population and treatment effect (p = 0.99), indicating that the effect of balanced crystalloids did not significantly differ between pediatric and adult patients (Figure 8).

A prespecified subgroup analyses were conducted based on fluid type, patient population, and DKA severity. There was no statistically significant difference between trials using Ringer’s Lactate (MD = −1.69 hours) versus Plasma-Lyte (MD = +2.00 hours) (Interaction p = 0.19), though the Plasma-Lyte subgroup relied on limited data. In a subset of patients identified with severe DKA (pH < 7.1 or bicarbonate < 5 mmol/L) from three trials, balanced crystalloids showed a trend toward a larger reduction in resolution time (MD = −3.50 hours; 95% CI: −11.01 to 4.01), but this was not statistically significant. When restricting the analysis to the four studies that used strict ADA-compliant composite definitions for DKA resolution (excluding Yung and Van Zyl), the effect size decreased (MD = −0.79 hours; 95% CI: −5.31 to 3.72), confirming that the inclusion of studies with purely biochemical endpoints did not drive the primary result.

### Publication Bias

**Figure. 9.**
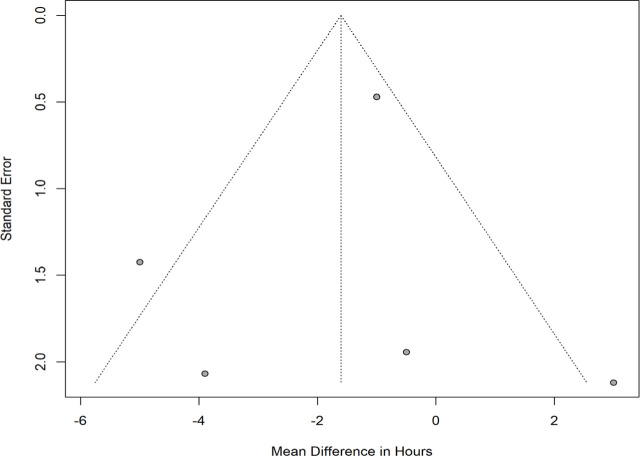
Funnel plot for the assessment of publication bias for the outcome of time to DKA resolution

The potential for publication bias for the primary outcome was assessed using visual and statistical methods as visual inspection of the funnel plot for “Time to DKA Resolution” revealed a reasonably symmetric distribution of studies around the pooled effect estimate, suggesting a low risk of small-study effects or publication bias (Figure 9) which was corroborated by a formal Egger’s regression test, which was not statistically significant (p = 0.78). These findings suggest that there is no evidence of publication bias for this outcome; however, this assessment is limited by the small number of included studies (k = 5), which reduces the statistical power of the test.

## Discussions

### Summary of Principal Findings

This SRMA synthesized evidence from 11 RCTs to compare the effects of balanced crystalloids versus normal saline for the initial fluid resuscitation of patients with DKA (Table 3). The primary finding demonstrated no statistically significant difference in the time to DKA resolution between patients treated with balanced crystalloids and those treated with normal saline. Although a trend favouring balanced crystalloids was observed, with an average reduction in the resolution time of 1.50 hours, the 95% confidence interval was wide and included the possibility of both benefit and harm. The analysis was constrained by the substantial and statistically significant heterogeneity among the studies included.

**Table 3.** GRADE Summary of Findings

**Outcome**	**Participants (Studies)**	**Effect (95% CI)**	**Certainty**	**Comments**
Time to DKA resolution	491 (6 RCTs)	MD-1.50 hrs (−3.79 to 0.79)	⨁⨁◯◯ Low	Downgraded for imprecision (wide CI) and inconsistency
Bicarbonate at 12h	230 (4 RCTs)	MD +2.50 mmol/L (1.51 to 3.48)	⨁⨁⨁◯ Moderate	Downgraded for imprecision (small sample size)
Acute kidney injury	357 (4 RCTs)	OR 0.84 (0.39 to 1.82)	⨁⨁◯◯ Low	Downgraded for imprecision (wide CI)

Among the secondary outcomes, balanced crystalloids resulted in a statistically significant and consistent improvement in serum bicarbonate levels at 12 hours (MD +2.50 mmol/L). Unlike clinical data, this biochemical signal was highly homogeneous (I^2^ = 0.0%). In addition, there was a trend towards a reduced incidence of hypokalemia with balanced crystalloids that approached statistical significance, while no significant differences were detected in other major clinical outcomes, including hospital length of stay, the incidence of AKI, or the odds of ICU admission.

### Interpretation of findings and comparison with existing evidence

The finding of this SRMA that balanced crystalloids do not significantly shorten the time to DKA resolution compared to normal saline highlights the persistent clinical equipoise on this topic, despite a clear physiological rationale favouring balanced solutions. The statistically significant improvement in serum bicarbonate levels observed at 12 h aligns with the lower chloride load, and the presence of buffer precursors in balanced crystalloids is expected to mitigate the development of hyperchloremic metabolic acidosis [12, 25]. This iatrogenic acidosis can mask the underlying correction of ketoacidosis, and its avoidance is the primary argument for using balanced fluids. This finding reflects the physiological difference in strong ion difference between fluids rather than a faster metabolic recovery from ketoacidosis per se. The bicarbonate improvement did not translate into a statistically significant reduction in the time to clinical DKA resolution.

The failure of this biochemical advantage to translate into a reduction in the primary outcome of overall DKA resolution is attributable to the substantial heterogeneity across the included trials was moderate (I^2^ = 36.2%). The prediction interval remains wide, suggesting that while the average effect is non-significant, the impact of balanced crystalloids may vary based on local protocols and patient severity.

The findings for secondary clinical outcomes are consistent with the lack of a benefit for the primary outcome, as the absence of a difference in hospital length of stay, incidence of AKI, and ICU admission suggests that the early biochemical differences observed with balanced crystalloids may not be of sufficient magnitude or consistency to alter the broader clinical course of most patients with DKA. The trend towards a reduction in hypokalemia (p = 0.057) is physiologically plausible, as the administration of chloride-rich, potassium-free normal saline can exacerbate urinary potassium losses and intracellular shifts, although this finding needs investigation in larger trials.

### Strengths of the review

This SRMA possesses methodological strengths that enhance the validity of its conclusions, as it is based on a protocol registered with PROSPERO, which minimizes the risk of reporting bias and ensures transparency. A search strategy was employed across multiple databases without initial language restrictions to maximize the capture of relevant studies. In addition, the methodological quality of the included trials was assessed using the revised Cochrane RoB 2 tool, providing an appraisal of the evidence base. Robust statistical methods were used for meta-analysis, including a random-effects model to account for heterogeneity and a pre-planned subgroup analysis to explore potential sources of inconsistency.

### Limitations

The most significant limitation was the substantial statistical and clinical heterogeneity observed across the included studies for the primary outcome due to variability in patient populations (pediatric vs. adult), differences in the severity of DKA at presentation, and variability in the definitions of DKA resolution across trials. While a sensitivity analysis was performed excluding non-standard definitions, the remaining pool of studies was small. Additionally, different balanced crystalloids (Ringer’s lactate vs. Plasma-Lyte) were pooled; while the subgroup analysis showed no interaction, the number of Plasma-Lyte studies was insufficient to prove equivalence.

The methodological quality of the primary studies was variable, as several included trials were open-label or had unclear blinding procedures, which introduced performance and detection biases. Moreover, some studies suffered from baseline imbalances between treatment groups, which could confound the results, or had low compliance with the assigned intervention, leading to treatment contamination. Another limitation is the relatively small number of studies available for each outcome, which restricts the power of the subgroup analyses and limits the reliability of the publication bias assessment.

### Implications for clinical practice and future research

The findings of this SRMA do not provide definitive evidence to recommend the routine use of balanced crystalloids over normal saline in all patients with DKA. There was a biochemical benefit in terms of a more rapid bicarbonate recovery and a potential reduction in hypokalemia, but this did not translate into a statistically significant improvement in the time to DKA resolution or other major clinical outcomes. As the choice of fluid remains a matter of clinical judgment and institutional preference and given the absence of a clear signal of harm alongside plausible physiological benefits, balanced crystalloids may represent a reasonable choice, particularly in patients expected to require large fluid volumes or in those with severe acidosis at presentation.

The substantial heterogeneity underscores the need for large, high-quality, and methodologically rigorous RCTs; therefore, future trials should aim to be double-blinded, powered for patient-important outcomes, and focus on more homogeneous populations (e.g., adults with severe DKA). Trials comparing different types of balanced crystalloids (e.g., Ringer’s lactate vs. Plasma-Lyte) are needed to determine whether a specific formulation offers superior benefits. Furthermore, an individual patient data (IPD) meta-analysis of existing trials could explore sources of heterogeneity and identify patient subgroups that might derive the greatest benefit from balanced crystalloids.

## Conclusion

This SRMA found that among patients with DKA, initial fluid resuscitation with balanced crystalloids did not result in a statistically significant reduction in the time to DKA resolution compared with normal saline. Although the use of balanced crystalloids was associated with a more rapid improvement in serum bicarbonate levels. However, it is crucial to note that this biochemical advantage did not translate into statistically significant clinical benefits, such as a reduction in time to DKA resolution or length of hospital stay. Owing to the substantial heterogeneity and methodological limitations of the existing evidence, the choice of crystalloid for initial DKA resuscitation remains an area of clinical equipoise, and further high-quality research is required to provide a definitive recommendation.

## Supplementary Material

Supplementary Material Details
