# Supplementary material for: Balanced crystalloids versus normal saline for initial fluid resuscitation in diabetic ketoacidosis: a systematic review and meta-analysis of randomized controlled trials
Source: J Crit Care Med (Targu Mures). 2026 Jul 27;12(3):328–41. doi: 10.62838/jccm-2026-0022 (PMC13403006; doi:10.62838/jccm-2026-0022)
Supplement: Supplementary file 1 — Supplementary Material Details [file jccm-2026-0022_Supplementary.pdf]

Table S1: Continuous Outcomes Data for Meta-Analysis

Outcome & Study	Intervention Group (RL/BC)	Control Group (NS)	Notes
	Mean	SD	N
**A. Time to DKA Resolution (hours)**			
Agarwal et al. (2025)	12.9	7.9	33
Self et al. (2020)	13.9	4.8*	94
Williams et al. (2020)	15.1	6.2*	34
Yan et al. (2024)	15.7	6.5*	25
Ramanan et al. (2021)	24.0	NR	48
Yung et al. (2017)	7.5	1.8	38
**B. Change in Serum Bicarbonate (mmol/L) at ~12 hours**			
Agarwal et al. (2025)	14.7	1.6	33
Mahler et al. (2011)	9.0	1.5*	22
Aditianingsih et al. (2017)	7.4	10.3*	15
Trifi et al. (2025)	15.4	4.1*	46
**C. Hospital Length of Stay (days)**			
Williams et al. (2020)	9.4	3.1*	34
Ramanan et al. (2021)	3.9	3.6*	48
Yan et al. (2024)	3.3	3.1*	25
Yung et al. (2017)	0.72	0.8*	38
**D. Total Fluid Volume Received (mL) until DKA resolution**			
Agarwal et al. (2025)	1184	464*	33
Attokaran et al. (2023)	2463	1270	46
Self et al. (2020)	4267	2045*	94
Yung et al. (2017)	2385	825*	38

Abbreviations: RL/BC: Ringer's lactate/balanced crystalloid (intervention); **NS:** 0.9% Normal Saline (control); **N:** Number of participants; **SD:** Standard Deviation; **AKI:** Acute Kidney Injury; **LOS:** Length of Stay; **NR:** Not Reported.

Table S2: Dichotomous Outcomes Data for Meta-Analysis

Outcome & Study	Intervention Group (RL/BC)	Control Group (NS)	Notes
	Events (r)	Total (N)	Events (r)
**A. Incidence of New or Progressive Acute Kidney Injury (AKI)**			
Agarwal et al. (2025)	0	33	2
Williams et al. (2020)	13	34	15
Self et al. (2020)	7	94	6
Yan et al. (2024)	4	25	3
**B. Incidence of Hypokalemia (<3.5 mmol/L)**			
Agarwal et al. (2025)	15	33	21
Self et al. (2020) [4]	9	94	15
Trifi et al. (2025)	NR	46	NR
Yan et al. (2024)	11	25	5
**C. ICU Admission**			
Attokaran et al. (2023)	18	46	19
Yan et al. (2024)	1	25	1
Self et al. (2020)	77	94	65

Abbreviations: RL/BC: Ringer's lactate/balanced crystalloid (intervention); **NS:** 0.9% Normal Saline (control); **N:** Number of participants; **SD:** Standard Deviation; **AKI:** Acute Kidney Injury; **LOS:** Length of Stay; **NR:** Not Reported.
